# *Darwininitium* – a new fully pseudosigmurethrous orthurethran genus from Nepal (Gastropoda, Pulmonata, Cerastidae)

**DOI:** 10.3897/zookeys.175.2755

**Published:** 2012-03-16

**Authors:** Prem B. Budha, Peter B. Mordan, Fred Naggs, Thierry Backeljau

**Affiliations:** 1 Central Department of Zoology, Tribhuvan University, Kirtipur, Kathmandu, Nepal; 2 University of Antwerp, Evolutionary Ecology Group Groenenborgerlaan 171, B-2020 Antwerp, Belgium; 3 Natural History Museum, Cromwell Road London, SW7 5BD, UK; 4 Royal Belgian Institute of Natural Sciences, Vautierstraat 29, B-1000, Brussels, Belgium

**Keywords:** Pseudosigmurethrous, Orthurethran, Pulmonata, *Darwininitium*, Nepal

## Abstract

A new genus and species of pseudosigmurethrous orthurethran pulmonate of the family Cerastidae, *Darwininitium shiwalikianum* **gen. n.** and **sp. n.** is described from the Lesser Himalaya of Nepal. It represents the first record of an orthurethran with a fully developed pseudosigmurethrous pallial system, having a completely closed secondary ureteric system. Biogeographically this new taxon provides a significant range extension for the family north of the previously known distribution range.

## Introduction

There are currently some 14 genera recognised in the orthurethran land snail family Cerastidae. Their greatest diversity is found in the Afro-tropical zone, but the family extends eastwards into the Indian subcontinent and beyond, and one genus, *Amimopina*, occurs in Australia and New Guinea (Solem 1964), Thailand (Sutcharit et al. 2010), and probably also Cambodia (Mordan 1992). A few species have become widely dispersed in many of the islands of the Indo-Pacific by human agency.

The family Cerastidae Wenz, 1923 was previously included in the Enidae sensu lato (Enoidea sensu Bouchet and Rocroi 2005), and first recognised as a distinct group of orthurethran land snails by Watson (1920: 22), who stated that there can be little doubt that *Pachnodus* and its allies should be placed in a separate subfamily from the Palaearctic forms, or perhaps even in a distinct family. The phylogenetic relationship of several cerastid genera was subjected to a morphology-based cladistic analysis by Mordan (1992), and the monophyletic nature of the family has recently been supported by molecular evidence (Wade et al. 2006).

The principal distinguishing anatomical feature of cerastids is the excretory system which, whilst generally being of the normal orthurethran form comprising an elongate kidney with a straight primary ureter running directly towards the pneumostome, has developed what Solem (1964: 115) has termed a ‘pseudo-sigmurethrous’ secondary ureter. This takes the form of a fold or tube running from the renal orifice back along the kidney to the top of the lung, and usually a partial or complete fold running back along the rectum, towards the pneumostome, the whole ureteric anatomy mirroring that of true sigmurethrous Stylommatophora. Partial or complete closure of the renal fold to form a tube is known in *Edouardia*, *Amimopina*, *Rachistia* and *Limicena* (Solem 1964, Mordan 1992, 1998), but none of these has a partly, let alone completely closed rectal fold. Presumably these anatomical structures have the effect of directing the excretory products of the kidney along a pathway in which there can be some water resorbtion, and are consistent with the group being broadly xerophilic in its habits.

**Figure 1.**
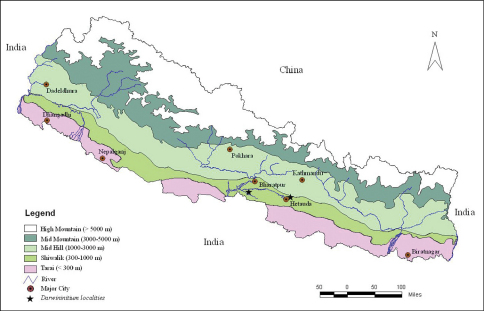
Map of Nepal showing the collection localities of *Darwininitium shiwalikianum* sp. n.

Here we present an account of a new genus and species of cerastid from the Lesser Himalaya of Central Nepal. Along its E-W axis, Nepal is divided into five east-west parallel physiographic elevational regions i.e. from south (lower) to north (higher): 1) Tarai (southern flat belt from 67 m – 300 m), 2) Shiwalik (from 300 m – 1000 m), 3) Mid Hill (1000 m – 3000 m), 4) Mid Mountain (3000 m – 5000 m) and 5) High Himalaya (> 5000 m). Our surveys were conducted in these five zones in Central Nepal but the new genus was reported only from the Shiwalik range (Figure 1). The type material has been deposited at the museum of the Central Department of Zoology, Tribhuvan University (CDZTU), Kathmandu, Nepal.

Shell measurements are expressed in mm as follows: shell height × max. shell width × min. shell width. Abbreviation for internal parts used are; AG- albumen gland, ATR- atrium, AU- auricle, E- epiphallus, EC- epiphallar caecum, GS- gametolytic sac, HD- hermaphrodite duct, KI- kidney, P- penis, PI- pilaster, PNE- pnerumostome, PR- penis retractor, REU- renal branch of secondary ureter, RT- rectum, RTU- rectal branch of secondary ureter, SO- spermoviduct, UO- ureteric orifice, VD- vas deferens and VE- ventricle.

## Taxonomic treatment

### Darwininitium

Budha & Mordan, 2012 gen. n.

urn:lsid:zoobank.org:pub:D670F9B7-1061-4686-B54B-54B4A1DBD4D5

#### Type species.

*Darwininitium shiwalikianum* Budha & Mordan, 2012, sp. n.

### Darwininitium shiwalikianum

Budha & Mordan, 2012 sp. n.

urn:lsid:zoobank.org:act:4C8FA14B-A9CA-49D6-A91A-B532B2EDBAEE

http://species-id.net/wiki/Darwininitium_shiwalikianum

#### Material.

 Holotype CDZTU0114: Kasara near Tamor Lake, Chitwan National Park, Central Nepal, ca 210 m. 27°61'10"N, 85°20'05"E, sal (*Shorea robusta*) forest, leg P.B. Budha 5 May 2008. Paratype from the type locality, CDZTU0114a/1 shell. Other paratypes CDZTU0115/2: Taubas along the road side of Tribhuvan Highway, Bhainse, left bank of Rapti river, Makwanpur [=district], Central Nepal, ca 520 m. 27°30'13"N, 85°02'59"E, 2 live. Mixed riverine forest, leg P.B. Budha 7 May 2008.

#### Diagnosis.

Cerastidae with a fully pseudosigmurethrous pallial system including fully enclosed renal and rectal branches of ureter; penial appendix lacking, which is present in all known cerastid genera. Shell carinated, white flecks on a dark-brown background.

#### Description:

**Figure 2.**
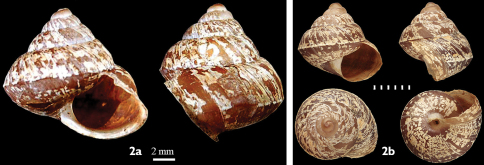
Shell of *Darwininitium shiwalikianum* sp. n. **2a** holotype CDZTU0114 **2b** paratype CDZTU0115

 Shell (Figure 2a, 2b): Dextral, globosely turbinate, weakly carinated, rimately perforate, whorls 5, apex blunt, suture shallow, chestnut with wide and irregular shaped radial white patches both in upper- and under- surface, first two whorls smooth but under magnification (60×) shallow dotted wrinkles visible, later whorls with weak radial striae, aperture ovate, peristome thin and reflected slightly descended toward the aperture, columellar margin reflected covering nearly half the umbilicus.

#### Shell dimensions

 (mm)**.** Holotype: 13.6 × 15.2 × 12.6, aperture 7.9 × 8.0, whorls 5, paratype from the type locality 14.1 × 13.8 × 12.5, whorls 5, aperture 7.8 × 7.9, paratypes from Taubas 11.7 × 12.5 × 10.6, aperture 6.9 × 7.5, whorls 4.5, 12.3 × 15.7 × 12.9, aperture 8.1 × 10.5, wh. 4.5.

#### Etymology.

 The genus is named as a tribute to the Darwin Initiative for having supported land snail projects and in particular for supporting the senior authors’ participation in the project Developing land snail expertise in South and Southeast Asia from 2006-2011. The species name derives from the Lesser Himalaya Shiwalik range, from where it was reported.

#### Animal

**Figure 3.**
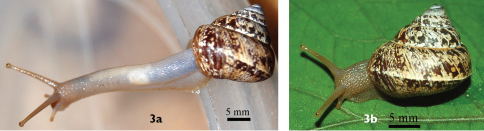
Live animal of *Darwininitium shiwalikianum*, paratype CDZTU0115 **3a** anterior portion extended **3b** animal *in situ*.

 (Figures 3a, 3b). The anterior of the uniformly pale cream body can extend significantly more than 2× of the shell length.

#### Pallial cavity

**Figure 4.**
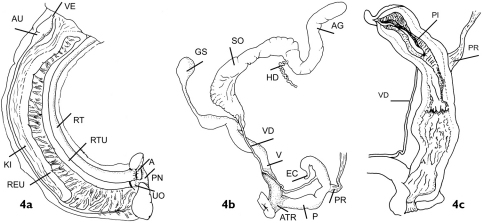
Genitalia in *Darwininitium shiwalikianum* sp. n. paratype CDZTU0115 **4a** Pallial cality **4b** General view of genitalia **4c** Interior of the penis

 (Figure 4a). Kidney of the typical orthurethran type, running approximately four-fifths the length of the pulmonary cavity; a thin-walled, completely closed tube runs from the renal pore along the full length of the inner margin of the kidney, folding at the top to run a short distance towards the rectum, and then down along the inner margin of the rectum, again as a fully closed tube, almost as far as the anus and close to the pneumostome, where it opens with a slight flare. Pallial venation is prominent and a mantle gland is lacking.

#### Reproductive system

 (Figures 4b, 4c). The female system has a well-developed gametolytic sac with a long peduncle with an expanded basal portion, and darkly pigmented spongy tissue in the atrium and at the base of the free-oviduct. The penis lacks an appendix, but has a prominent epiphallar caecum. The penial retractor inserts well below and opposite the point of insertion of the vas deferens. Internally the penis is separable into two regions, the lower having numerous longitudinal pilasters with a knobbly appearance. Above there is a slight constriction of the lumen by an undulating ring pilaster with a smooth, forked pilaster above, running downwards from the level of the opening of the vas deferens. The epiphallus has a thick pad of transverse, weakly ridged tissue running down its length more-or-less opposite the pore of the vas deferens, as well as a smooth longitudinal fold which runs down from within the caecum as far as the top of the vas deferens opening. There is no obvious penial sheath. The hermaphrodite duct lacks the clump of diverticulae characteristic of the Enidae sensu stricto.

#### Distribution.

**Figure 5.**
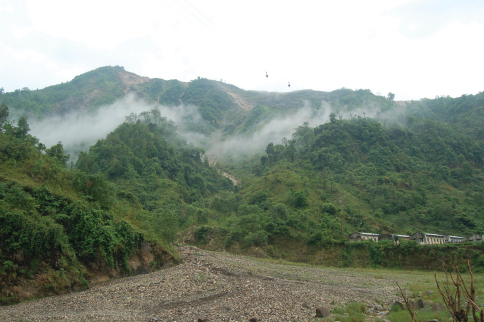
Habitat of *Darwininitium shiwalikianum* sp. n.

 The genus *Darwininitium* was collected in the Dun valley of the Lesser Himalaya in the Rapti river basin from an elevation of ca 210 m in Chitwan National Park and extending northward to Taubas, Bhainse Makwanpur, Central Nepal at an elevation of ca 520 m above sea level. The area has a humid, sub-tropical climate and comprises sandstones, siltstones and mudstones. The forest is dominated by tropical sal (*Shorea robusta*) mixed with *Terminalia* sp. in the national park, and mixed riverine forest with major tree species of *Acacia catechu*, *Dalbergia sissoo*, *Bombax ceiba* along the river belt at Bhainse (Figure 5).

## Discussion

*Darwininitium* is of particular interest on two counts: firstly its distribution represents a considerable extension of the known range of the Cerastidae, and secondly it exhibits the most advanced development of the pseudosigmurethrous condition yet found in the family, or indeed in any orthurethran species.

A very short partial renal fold is found in some genera in a few families of Orthurethra, for example *Acanthinula* (family Acanthinulidae), but a secondary ureteric structure is by far the most clearly and widely developed in the Cerastidae. Pseudosigmurethry was first described in the Australian cerastid *Amimopina macleayi* (Brazier) by Solem (1964). This monotypic genus has a fully closed renal tube running along the side of the kidney, from the renal pore to almost the top of the lung. It terminates as a short, so-called ‘renal ridge’. There is a short open gap across the top of the lung cavity before a rectal fold, open along its entire length, runs almost the full length of the rectum as far as the pneumostomal complex. In *Darwininitium* the rectal fold is fused to the rectum along its entire length, meaning that there is a continuous tube running from the tip of the kidney to its opening adjacent to the anus and pneumostome, mirroring more closely than previously known the situation in the true Sigmurethra.

The position of *Darwininitium* within the Cerastidae is confirmed by the well-developed pseudosigmurethry, as well as by the highly characteristic darkly pigmented spongy tissue lining the atrium, not known in any other orthurethran family, and the prominent pallial venation (Mordan 1992). It differs from the Enidae, in which the family was earlier included, in lacking hermaphrodite duct diverticulae and a mantle gland.

In addition to its unique pallial anatomy, *Darwininitium* is immediately separable from other cerastid genera for which there is anatomical information, by the absence of a penial appendix: an elongate, tubular structure, highly differentiated into several distinct regions along its length, inserting well below the point of insertion of the vas deferens, and with its own retractor muscle system. The appendix is found in all other cerastid genera, and occurs commonly throughout the Orthurethra. Indeed, it is difficult to associate *Darwininitium* phylogenetically with any of the existing cerastid genera. Mordan (1992, 1998) has analysed the anatomy of the reproductive, alimentary and pallial systems of 11 genera of cerastid and produced a morphological phylogeny based largely on these characters. One clade, which includes *Rhachistia*, *Edouardia*, *Amimopina* and *Limicena*, has a renal fold which is either partly or wholly fused along its length to form a tube, and therefore most closely approaches the condition found in *Darwininitium*, but none of these shows any fusion of the rectal fold, let alone complete fusion in the form of a tube along its entire length. However, this clade is further characterised by an extremely short gametolytic duct, and a relatively long and very narrow flagellum which inserts on the caecum at the head of the penis, level with or above the point of insertion of the vas deferens, characters not found in *Darwininitium* which has a rather long gametolytic duct and no flagellum. The shell of *Darwininitium* is closest in shape to certain carinated species of *Edouardia*, but the pigmentation of white flecks on a dark-brown background is unlike any other cerastid.

Biogeographically this new record provides a significant range extension to the family Cerastidae, Nepal lying significantly further north of the previous known distribution. Mordan (1992; Fig. 1) summarised the distribution of the Cerastidae, which is centred on the Afro-tropical region with by far the greatest diversity being found in southern Africa and Arabia. There are outliers in the Indian subcontinent, Cambodia, Thailand, Australia, South-Eastern New Guinea, and now Nepal. Additionally a few species such as *Rhachistia histrio* (Pfeiffer) have spread into many other islands of the Indo-Pacific, and were considered by Solem (1959) at least in some cases to have been dispersed by human agency. In the Indian subcontinent cerastids are restricted to a band running down the west coast and including Sri Lanka, and an area around the Ganges delta.

The detailed anatomy of the Australian species, *Amimopina macleayi*, was described by Solem (1964). Mordan (1992) postulated that *Bulimus subangulatus* Pfeiffer from the Lao Mountains, Cambodia, was a species of *Amimopina*, based on the two shells in the syntype series in the Natural History Museum, London. Since then, Sutcharit et al. (2010) have recorded *subangulatus* from Thailand, along with two species of *Rhachistia*, and have shown *subangulatus* to be an *Amimopina* on the basis of dissection. We are thus gaining ever more detailed information on the continental distribution of the Cerastidae in the Australasian region, and this is turning out to be far less disjunct than previously thought.

## Supplementary Material

XML Treatment for Darwininitium

XML Treatment for Darwininitium shiwalikianum
